# Evaluating Healthcare Worker Competence in the Correct Use of Personal Protective Equipment in the COVID Era: A Quality Improvement Project

**DOI:** 10.7759/cureus.11954

**Published:** 2020-12-07

**Authors:** Max Prokopenko, Maria Katerina Pissaridou, Omar Risk, Harman Khatkar

**Affiliations:** 1 Trauma and Orthopaedics, Oxford University Hospitals NHS Foundation Trust, Oxford, GBR; 2 Research, Aceso Global Health Consultants Ltd, London, GBR; 3 Emergency Medicine, Milton Keynes University Hospital NHS Foundation Trust, Oxford, GBR; 4 Trauma and Orthopaedics, Royal Berkshire Hospital, Reading, GBR; 5 Nuffield Department of Orthopaedics, Rheumatology and Musculoskeletal Sciences, University of Oxford, Oxford, GBR

**Keywords:** covid-19, covid-19 pandemic, public health and safety, covid 19, personal protective equipment, healthcare worker safety, patient safety

## Abstract

Introduction: Throughout the coronavirus disease 2019 pandemic, personal protective equipment (PPE) guidance has rapidly evolved. Healthcare workers (HCWs) should use PPE correctly to reduce the risk of nosocomial transmission of the coronavirus. We predict a lack of training regarding correct PPE usage amongst HCWs and introduce a low-resource method of training.

Methods: HCWs from various disciplines at a District General Hospital self-rated their ability in utilising PPE using uncontrolled pre- and post-session 16-item questionnaires following a single PPE training session. Participant responses were analysed using Student's t-test for independent (unpaired) samples.

Results: Of 64 participants, 37 participants (59%) received any prior PPE training. Six participants (9%) previously received specific severe acute respiratory syndrome coronavirus 2 PPE training. Survey scores were higher in the post-test than the pre-test group.

Conclusion: This study highlights the lack of formal PPE training amongst HCWs and the need for establishing PPE training as part of the mandatory training of HCWs.

## Introduction

Coronavirus disease 2019 (COVID-19) is a viral disease caused by severe acute respiratory syndrome coronavirus 2 (SARS-CoV-2). It was detected in humans at the end of 2019, in Wuhan, Hubei, China [1]. Although not stated definitively, it is believed that SARS-CoV-2 originated from an animal host in the Huanan market of Wuhan, with zoonotic animal-to-human transmission [1]. Subsequent sustained human-to-human transmission has resulted in a pandemic, announced by the World Health Organization on 11 March 2020, with unprecedented socioeconomic and health implications [2]. The spectrum of clinical manifestations of COVID-19 ranges from asymptomatic to severe respiratory failure, necessitating invasive ventilation and organ support [3]. Preliminary data suggests infection with SARS-CoV-2 can stimulate an immune reaction, with a proliferation of immune factors leading to a ‘cytokine storm’. The result of this is extensive tissue damage [3]. Human-to-human transmission occurs predominantly via direct contact with infected cases and respiratory droplets (>5 μm) [4]. These are generated from the upper respiratory tract secretions of infected patients and dispersed by coughing or sneezing [5]. The maximum range from an infected source at which infection is possible has not been formally established; however, an area of one meter surrounding the infected person is thought to pose significant risk [6]. Aerosol generating procedures (AGPs) producing particles ≤5 μm, such as endotracheal tube intubation or tracheostomy insertion, are regarded as higher risk for transmission due to exposure to aerosols, increasing the risk of viral transmission to staff [7]. To protect healthcare workers (HCWs), Public Health England (PHE) has advised measures to mitigate this risk at healthcare institutions across the United Kingdom [2]. PHE recommends type IIR compliant fluid-resistant surgical masks (FRSM) to be worn in all clinical areas to reduce risk of droplet transmission [3].

Given the new and rapidly evolving guidance surrounding personal protective equipment (PPE) both at local and national levels, we hypothesise a lack of prior formal training, experience, and knowledge amongst HCWs in its correct utilisation. HCWs are unlikely to have had formal training in accordance with new guidelines and may consequently be at risk of nosocomial transmission. We propose the need for the rapid training of HCWs in safe PPE usage. This study aims to demonstrate the feasibility of implementing widespread training of HCWs in order to achieve competency. To avert nosocomial spread, PPE must be used correctly and consistently by HCWs in the healthcare setting. Poor compliance in PPE usage has previously been illustrated amongst HCWs [8]. Incorrect PPE application or subsequent removal, termed “donning” and “doffing”, puts HCWs at risk of exposure and infection [9]. Phan et al. demonstrated that 90% of all observed doffing practices recorded by observing HCWs in their study were incorrect [9]. Errors were made with regard to the doffing sequence and technique as well as selection of appropriate PPE. Inconsistencies in doffing therefore put HCWs at risk of infection through self-inoculation by contact between infected materials and their skin or clothes. Prior studies have demonstrated that infection of HCWs with high consequence infectious diseases (HCIDs), such as Ebola virus, may be because of incorrect PPE use [10].

Multiple factors are likely to contribute to errors in PPE usage [11]. Modifiable factors include the lack of knowledge and skills that may be amenable to improvement through practice and skills training. Phan et al. confirmed from their observations that participants were not familiar with the protocol surrounding PPE usage. Formal training builds both knowledge and skills, and consequently may promote correct PPE usage by HCWs as a consequence of having a more comprehensive understanding of PPE [9].

We predict that many HCWs have not had prior formal training in correct PPE usage and require training to reduce the risk of nosocomial transmission. This study investigates whether HCWs at a local level are adequately trained in PPE usage in relation to SARS-CoV-2. To our knowledge, this is the first study in the United Kingdom of its kind and introduces a quick and resource-effective method of training.

## Materials and methods

This is a pre-post study comparing outcomes in a cohort of HCWs before and after a single PPE training session. We predict a lack of knowledge and technical ability surrounding correct PPE practices amongst HCWs. We aimed to gauge the level of understanding and ability of correct PPE usage at a single District General Hospital (DGH) and a facility funded by a private healthcare provider in the United Kingdom. All training was carried out within this healthcare setting.

Study setting

The DGH site in this study has over 200 inpatient beds, serving a catchment population of 150,000, treating around 120,000 inpatients each year. Services offered by this hospital include an Emergency Department, Acute General Medical services, Trauma & Orthopaedics, Maternity services, Paediatrics as well a host of specialist outpatient clinics run by visiting consultants from the nearby tertiary teaching hospital.

Participants

**Figure 1:**
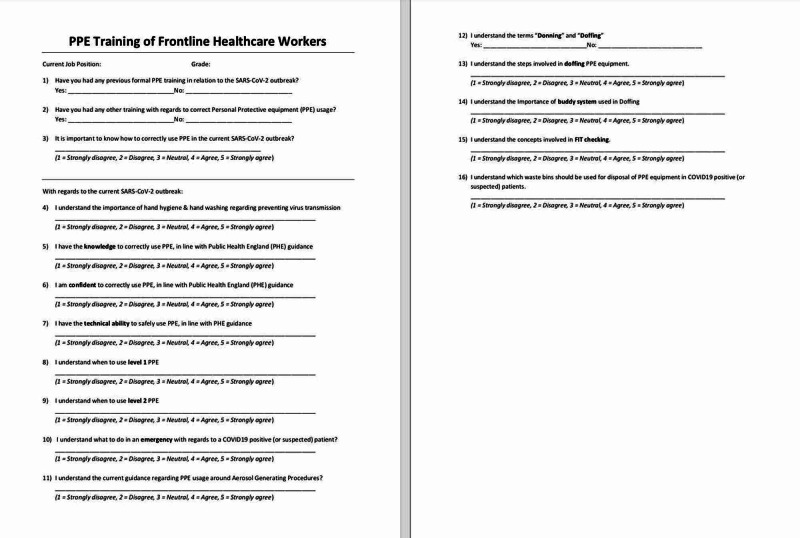
The contents of questionnaire used to collect data PPE, personal protective equipment; COVID19, coronavirus disease 2019; SARS-CoV-2, severe acute respiratory syndrome coronavirus 2

All participants (surgeons, doctors, nurses, allied health professionals and administrative staff attending PPE training sessions) were eligible for inclusion into this study by completion of study questionnaires before and after training. A paper questionnaire was used to collect data from all participants attending for PPE training (Figure 1).

All data was collected prospectively. All participants completing both parts of the questionnaire, prior and following training, were included in the study. Participants who failed to complete either part of the questionnaire were excluded. Data was collected over multiple PPE training sessions spanning a period of two weeks in March 2020. Given that evolving guidance on PPE usage could influence participant perception surrounding PPE, we ensured to train all participants within the shortest time frame possible in order to minimise any such potential effect.

Intervention

A team of three doctors, specifically trained in providing PPE training, delivered all PPE training in person via seminar-based sessions. Teaching utilised local protocols in the form of a PowerPoint presentation derived from official PHE guidance regarding donning and doffing techniques for level 1 and 2 PPE [12]. Sessions were limited to 10 participants. A total of eight sessions were delivered. Sessions covered modes of transmission, the types and purpose of the PPE available, as well as infection control strategies relating to SARS-CoV-2. We acknowledge these guidelines are rapidly evolving, and consequently advised all participants guidance may continue to change. Participants were required to demonstrate correct PPE usage under direct observation from a formally trained medical professional in accordance with up-to-date PHE guidance, which was printed and used as a protocol. Participants were directly observed to ensure correct application and removal of PPE was performed to a safe standard for participants to be certified as competent at the end of each session.

We assessed trainees via questionnaire immediately before and following training. Utilising a 16-item questionnaire, we asked participants to self-rate their knowledge, confidence and perceived ability of using PPE correctly, via a five-point Likert scale (strongly disagree, disagree, neutral, agree, strongly agree). Additionally, we ascertained the professional background and grade of participants, as well as whether they had any prior PPE training at all, or in relation to the current SARS-CoV-2 outbreak. Questionnaires were made anonymous to ensure responses could be made in confidentiality.

Participants’ questionnaires were tabulated using Microsoft Excel and analysed using Stata 16 (StataCorp, College Station, TX). Participant characteristics were summarised (role, grade, and previous PPE training). Each Likert item was dichotomised to “strongly disagree, disagree or neutral” scoring 0 versus “agree or strongly agree” scoring 1. The total number of items to which each respondent had agreed was calculated. The distribution of this number was visually inspected with a histogram. The mean of this number was compared between pre- and post-training groups using Student’s t-test for independent (unpaired) samples. Although this data is not normally distributed, this test is robust to violations of the parametric assumption. Although the data is dependent, a test for independent samples is valid but will have lower power as compared with a paired t-test. A paired t-test was not possible because a key linking pre- and post-training responses was not retained.

The study was registered with the regional audit approval service. Ethics approval was not required for this study. Written consent for study inclusion was obtained from all study participants.

## Results

There were 69 participants who underwent training in eight sessions. The response rate was 100%. Of these, five gave incomplete responses and were excluded. Table 1 shows sample characteristics. The sample consisted of 64 participants. Of these, 27 were nursing staff (42%), 21 allied health professionals (33%), 6 surgical doctors (9%), 5 administrative staff (8%), and 5 medical doctors (8%). Of these, 46 (72%) were junior and 18 (28%) were senior. The majority [n=37 (59%)] had received some form of PPE training in the past. Of these, 6 (9%) had received PPE training in relation to COVID-19.

**Table 1:** Sample characteristics PPE, personal protective equipment; COVID-19, coronavirus disease 2019

Characteristics		N (%)
Specialty		
Allied healthcare professional		21 (32.8%)
Administrative staff		5 (7.8%)
Medical doctors		5 (7.8%)
Nursing		27 (42.2%)
Surgical doctors		6 (9.4%)
Grade		
Junior		46 (71.9%)
Senior		18 (28.1%)
Any previous PPE training		
No		26 (41.3%)
Yes		37 (58.7%)
COVID-19 specific previous PPE training		
No		58 (90.6%)
Yes		6 (58.7%)
PPE importance		
Strongly disagree to neutral		5 (7.8%)
Agree or strongly agree		59 (92.2%)

**Figure 2:**
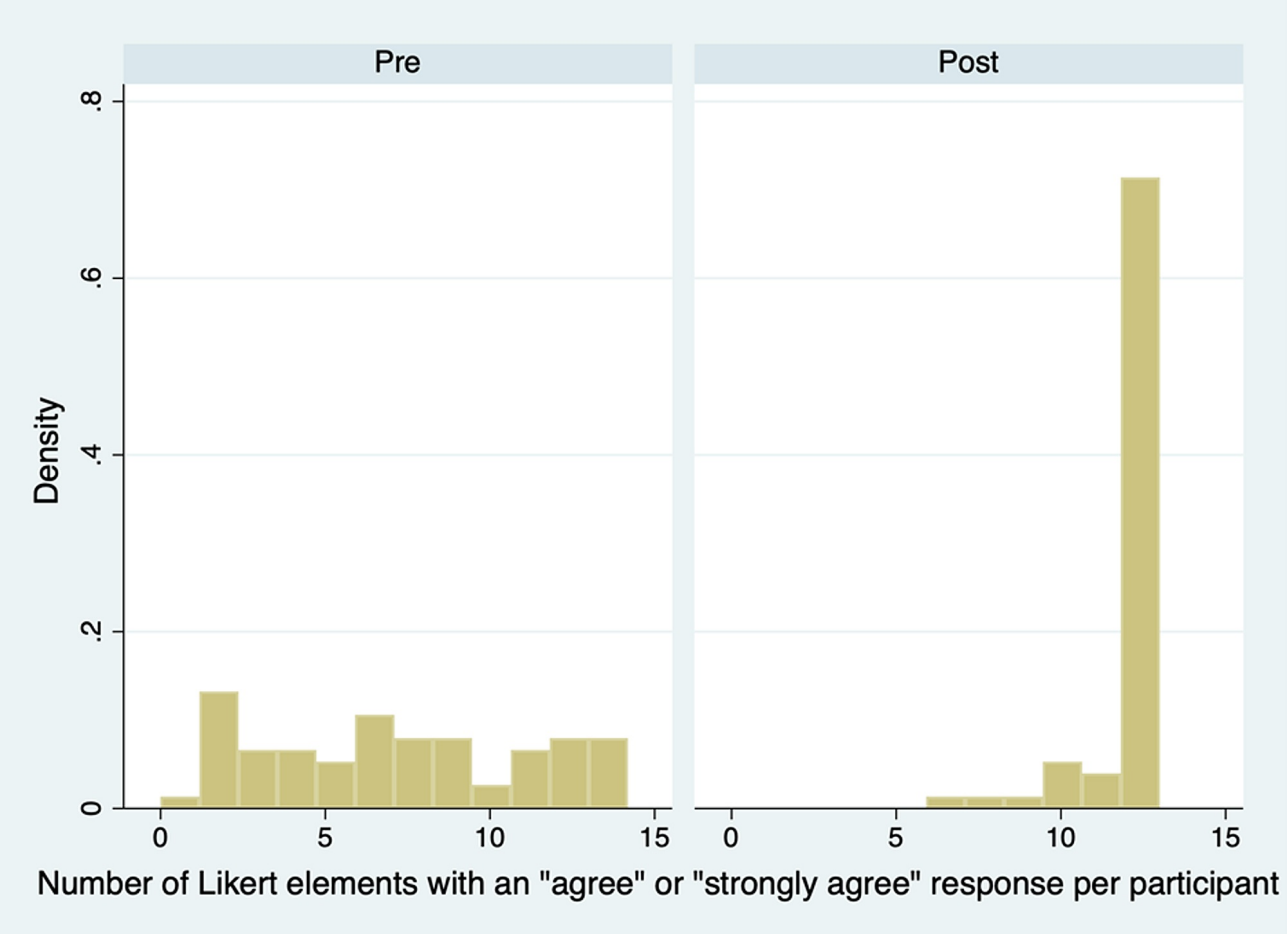
Histogram of the number of agreed responses per participant in pre- and post-training groups

Figure 2 shows the distribution of the number of agreed questions per participant in pre- and post-training groups. Visual inspection suggests an increase in agreement. The mean number of agreed questions in the pre- and post-training test surveys was 7.06 (95% CI 6.09-8.02) and 11.6 (95% CI 11.4-11.9), respectively. Student’s t-test for independent samples of the null hypothesis, that the means in pre- and post-test groups were equal, was statistically significant at the 95% confidence level (p-value < 0.0001). The difference in means was 4.58 (3.59-5.56). Without a control group for comparison, this result should be interpreted cautiously, and we should not conclude that this increase was necessarily because of our intervention.

## Discussion

We believe this is the first study of its kind to evaluate the understanding of correctly using PPE within the UK healthcare infrastructure. The study findings demonstrate some participants had prior training in PPE usage, but few had formal training of correct PPE usage specifically in relation to COVID-19. We demonstrate that a single training session can improve self-reported knowledge, confidence, and ability surrounding correct PPE usage across the wider multidisciplinary team.

These results raise critical questions surrounding PPE understanding within UK healthcare provision. Before COVID-19, HCWs were continuously exposed to communicable diseases that required understanding of, and ability to use PPE in order to prevent spread. Previous pandemics, including the SARS-CoV-1 outbreak in 2002 and H1N1 influenza outbreak in 2009, resulted in a significant loss of human life and societal disruption [13]. The UK Influenza Pandemic Preparedness Strategy 2011, produced in response to the aforementioned outbreaks, discusses the need for the adequate provision of PPE, but does not allude to effective training in the application of PPE [14]. Our study demonstrates clearly that a significant number of participants had no formal training prior to the sessions we delivered.

Given this lack of formal training combined with the risk posed by COVID-19, we believe there is a national requirement for widespread and rapid training of all HCWs in the correct usage of PPE to achieve competency.

In our experience, the training methods employed in this study have improved the safety and competency of PPE usage amongst a broad range of HCWs and has done so within a short time frame. Training appeared effective and was associated with minimal resource expenditure - primarily focused upon utilisation of PPE equipment and subsequent waste disposal, with trainers delivering sessions on a voluntary basis.

The lack of previous PPE training in our cohort also raises the question of whether adequate preparation is provided prior to commencing work in a clinical environment for all patient facing roles. For example, the General Medical Council’s (GMC) “Outcome for Graduates” clearly states that medical graduates should correctly use personal protective equipment (for example, gloves, gowns and masks) [15]. The findings from this study suggest that there is a clear discrepancy between perceived learning outcomes of the GMC and the actual understanding of PPE amongst practicing clinicians. Whether this is an issue of inadequate undergraduate education or de-skilling post-graduation is beyond the scope of this current study and requires further work.

Limitations

Data was collected from two different healthcare institutions, a district general hospital and a hospital funded by a private healthcare provider. To more fully evaluate the understanding of PPE amongst HCWs in the COVID era, a greater quantity of representatives from primary, secondary and tertiary care, in a wide variety of geographical settings, could be employed. Furthermore, we were limited to evaluating participants from ward and theatre-based environments in a secondary care setting. Future studies should involve HCWs across different clinical environments, for example, inclusive of intensive care and laboratory settings. The assessment of training delivered in this study remained formative, without a clear, objective summative assessment. Additionally, we did not follow up the participants to gauge whether the new knowledge and skills were retained in practice. Further studies should evaluate participant competence following a period after training.

In order to prevent nosocomial transmission, it is critical to understand and correctly employ infection control and prevention strategies, including the ability to don and doff PPE. We feel future PPE training should involve an element of competency-based assessment. Currently there is no universal, robust assessment or validated tool for discerning whether HCWs trained in using PPE are able to do so to a safe standard whilst caring for COVID patients, nor is there a consensus surrounding the most effective training method [16]. The Ebola outbreak that began in 2014 required strict adherence to infection prevention and control practices, owing to the severity of disease once transmission was successful [17]. Extrapolating and adapting practices from previous outbreaks such as the aforementioned should be approached with caution, given each outbreak represents unique challenges relating to transmission, case fatality ratio and overall epidemiological dynamics [18].

Furthermore, in order to validate and ensure the critical success of PPE training going forward, we propose two strategies. Strategies could include summative assessment in a prescribed period after the initial training and additionally a refresher course, alongside an update on best practice. We believe PPE training should be part of mandatory annual teaching for all HCWs.

## Conclusions

In the midst of the COVID-19 pandemic, it is apparent from the work of this study that the approach to understanding the correct use of PPE is a more reactionary measure, seeking to mitigate for knowledge gaps during the SARS-CoV-2 outbreak. Given historical pandemics and the well-documented need for accurately applied PPE, pandemic preparedness within the scope of this study appears to be lacking the appropriate emphasis on PPE training. PPE training should be a continuous fixture in the mandatory training of HCWs, with regular assessment and adaptation of practices in accordance with the latest evidence base. Future recommendations based on this research include adapting standard operating procedures in order to accommodate this, employing regular summative assessments. This would serve to provide a faster and more coordinated effort in response to preventing widespread transmission.
